# Triple Combination of Ascorbate, Menadione and the Inhibition of Peroxiredoxin-1 Produces Synergistic Cytotoxic Effects in Triple-Negative Breast Cancer Cells

**DOI:** 10.3390/antiox9040320

**Published:** 2020-04-16

**Authors:** Malgorzata Bajor, Agnieszka Graczyk-Jarzynka, Katsiaryna Marhelava, Malgorzata Kurkowiak, Arman Rahman, Claudia Aura, Niamh Russell, Agata O. Zych, Malgorzata Firczuk, Magdalena Winiarska, William M. Gallagher, Radoslaw Zagozdzon

**Affiliations:** 1 Department of Clinical Immunology, Medical University of Warsaw, Nowogrodzka 59, 02-006 Warsaw, Poland; malgorzata.bajor@wum.edu.pl (M.B.); k.marhelava@gmail.com (K.M.); 2 Department of Immunology, Medical University of Warsaw, Nielubowicza 5, 02-097 Warsaw, Poland; agnieszka.graczyk-jarzynka@wum.edu.pl (A.G.-J.); a_zych@interia.pl (A.O.Z.); mfirczuk@wum.edu.pl (M.F.); magdalena.winiarska@wum.edu.pl (M.W.); 3 Postgraduate School of Molecular Medicine, Medical University of Warsaw, Trojdena 2a, 02-091 Warsaw, Poland; 4 International Centre for Cancer Vaccine Science, University of Gdansk, Wita Stwosza 63, 80-308 Gdansk, Poland; mszczepaniak86@gmail.com; 5 Cancer Biology and Therapeutics Laboratory, UCD School of Biomolecular and Biomedical Science, UCD Conway Institute, University College Dublin, D04 Dublin 4, Ireland; arman.rahman@ucd.ie (A.R.); claudia.auragonzalez@ucd.ie (C.A.); niamh.russell@ucd.ie (N.R.); william.gallagher@ucd.ie (W.M.G.); 6 OncoMark Ltd., Nova UCD, D04 Dublin 4, Ireland; 7 Department of Immunology, Transplantology, and Internal Diseases, Medical University of Warsaw, Nowogrodzka 59, 02-006 Warsaw, Poland

**Keywords:** triple therapeutic combination, antioxidant enzyme, prooxidant agents, augmented prooxidant therapy, triple-negative breast cancer

## Abstract

Triple-negative breast cancer (TNBC) is an aggressive form of mammary malignancy currently without satisfactory systemic treatment options. Agents generating reactive oxygen species (ROS), such as ascorbate (Asc) and menadione (Men), especially applied in combination, have been proposed as an alternative anticancer modality. However, their effectiveness can be hampered by the cytoprotective effects of elevated antioxidant enzymes (e.g., peroxiredoxins, PRDX) in cancer. In this study, PRDX1 mRNA and protein expression were assessed in TNBC tissues by analysis of the online RNA-seq datasets and immunohistochemical staining of tissue microarray, respectively. We demonstrated that PRDX1 mRNA expression was markedly elevated in primary TNBC tumors as compared to non-malignant controls, with PRDX1 protein staining intensity correlating with favorable survival parameters. Subsequently, PRDX1 functionality in TNBC cell lines or non-malignant mammary cells was targeted by genetic silencing or chemically by auranofin (AUR). The PRDX1-knockdown or AUR treatment resulted in inhibition of the growth of TNBC cells in vitro. These cytotoxic effects were further synergistically potentiated by the incubation with a combination of the prooxidant agents, Asc and Men. In conclusion, we report that the PRDX1-related antioxidant system is essential for maintaining redox homeostasis in TNBC cells and can be an attractive therapeutic target in combination with ROS-generating agents.

## 1. Introduction

Triple-negative breast cancer (TNBC) is one of the most aggressive forms of mammary malignancies with dismal prognosis due to the shortage of effective treatment. While some accomplishments have recently been made with the combination of chemotherapy and immune checkpoint blockade, the overall effectiveness of such treatment is still far from satisfactory [1]. Therefore, a continual need exists for more successful therapies in TNBC.

Prooxidant therapies have been proposed for several decades as a promising alternative to classical anticancer chemotherapy. The principle of such approaches relies on the evidence that, due to metabolic dysfunction in redox homeostasis, cancer cells generate excessive amounts of reactive oxygen species (ROS) that, if further exaggerated by ROS-generating pharmaceutics, can induce cancer-specific cell death [2]. Based on this assumption, many prooxidant agents have been studied in preclinical settings, and some of these compounds entered clinical trials, alone or in combination. One of the most promising combinations has been the concomitant application of high doses of ascorbate (L-ascorbate, a reduced form of vitamin C, Asc) and menadione (vitamin K3, Men) [3]. When applied together, these two agents induce an abrupt and potent generation of ROS in cancer cells that have proved cytotoxic across a range of malignancies [4,5,6,7]. Based on these observations, a clinical phase I/IIa study in prostate cancer patients was conducted in the late 2000s [8]. However, despite high expectations, the orally administered Asc/Men combination produced only modest benefits for cancer patients [8], which hampered further trials with this therapy in clinical settings. One of the most probable causes for the inferior effects of Asc/Men combination in humans in comparison with the laboratory settings is the adaptation of cancer cells to suboptimal concentrations of the prooxidants by increasing their natural antioxidant defenses. The failure to achieve the optimal systemic concentrations of Asc and Men originates from the poor bioavailability of these compounds administered orally [9,10]. Perhaps the intravenous infusion of Asc (reviewed in [11]) combined with intravenous Men [12] could be a solution, but such treatment has not been evaluated in a properly designed randomized trial in humans up to date. The other type of approach, as reported in the current work, is to specifically sensitize cancer cells to the lower concentrations of Asc and Men by blocking the antioxidant defenses.

As mammalian cells harbor multiple defense mechanisms responsible for maintaining the redox homeostasis (e.g., catalase, glutathione peroxidases, or peroxiredoxins), it is crucial to identify which of the intracellular antioxidant systems can be responsible for protecting the cancer cell against Asc/Men-induced toxicity. Indeed, the previous research from our team and others suggested that the effectiveness Asc [13] or Men [14] against cancer cells can be dramatically increased by genetic or chemical inhibition of peroxiredoxin 1 (PRDX1). Importantly, when a close homolog of PRDX1, i.e., PRDX2, was genetically targeted, there was no amplification of Asc-induced toxicity against breast cancer cells [13]. This suggests a superior role for PRDX1 in defending the mammalian malignancies from the consequences of prooxidant treatment.

PRDX1 belongs to the family of six mammalian peroxiredoxins [15], with most of them (PRDX1–5) acting as antioxidants in an enzymatic chain with thioredoxin (TXN) and thioredoxin reductase (TXNR). However, as mentioned above, experimental evidence suggests that PRDX1 can perform exceptional antioxidant functions in comparison with other peroxiredoxins. Indeed, while genetic knock-outs of other peroxiredoxins do not significantly hamper the survival of the animals and cause relatively mild defects [16,17,18,19,20], *Prdx1*-deficient mice suffer from shortened survival due to development of hemolytic anemia and multiple tumors, including mammary carcinomas [20]. The role of PRDX1 during the development of breast cancer is, however, complex. The previous research from our group and others suggests that PRDX1 protein is upregulated in breast cancer as compared to the non-malignant mammary cells [13,21], and, by stabilizing the redox balance, it can inhibit the progression of breast cancer into more aggressive forms [13,22]. Thus, while PRDX1 upregulation is a biomarker of poor prognosis in a range of human malignancies, increased expression of PRDX1 in estrogen receptor-positive breast cancer has been shown to correlate with favorable prognosis [22]. Simultaneously, however, PRDX1 can protect breast cancer cells from the effects of certain forms of treatment [23,24], including prooxidant compounds [13], and therefore can be regarded an attractive target for anticancer therapies in mammary malignancies. In this study, we evaluated the applicability of targeting PRDX1 along with the Asc/Men combination as a synergistic antitumor approach in a TNBC model context. Our data clearly suggest further amplification of the cytotoxic effects by such triple combination specifically against TNBC, but not against the non-malignant mammary cells.

## 2. Materials Methods

### 2.1. Analysis of RNA-seq Data

Gene expression values (fragments per kilobase of transcript per million reads, FPKM) were downloaded from the NCBI Gene Expression Omnibus (GEO) database (accession number GSE58135). FPKM values were derived from the Cufflinks 1.3.0 software, run with the -u option, as described in [25]. Expression values of the *PRDX1* gene for the triple-negative breast cancer (TNBC, *n* = 42) and uninvolved breast tissue samples that were adjacent to the TNBC primary tumors (*n* = 21) were retrieved from the whole dataset. *PRDX1* presented FPKMs above 1 in all analyzed samples. The expression values in the two above-mentioned groups were presented on a boxplot in log2 scale using ggplot package in *R*, and the *p*-value between the groups was calculated in *R* using Welch two-sample *t*-test.

### 2.2. TMA Cohort

The RATHER TNBC tissue microarray (TMA) cohort contains formalin-fixed paraffin-embedded (FFPE) tissues from 138 TNBC patients. In this study, 109 TNBC patient samples censored at 15 years were analyzed. Ethical approval for materials used as part of the RATHER project was previously obtained from relevant committees in the Netherlands Cancer Institute and Cambridge University. The analyzed TMA cohort has been described previously in detail [26].

### 2.3. Immunohistochemistry

Immunohistochemistry (IHC) stainings of TMA sections were performed using an automated IHC platform (Link-48, Dako, Glostrup, Denmark) as described previously [27]. A polymer-based detection system (EnVision Flex, Dako Agilent, CA, USA) was used with 3,3′-diaminobenzidine (DAB) as the chromogen, resulting in a brown color endpoint. Sections were counterstained with hematoxylin. The TMA sections were stained with anti-PRDX1 antibody (cat. No. HPA007730, Sigma-Aldrich, St. Louis, MO, USA, dilution 1:150) as described previously [21]. Positive and negative controls (omission of the primary antibody and replacement with the rabbit-IgG isotype control (cat. No. ab208334, Abcam, Cambridge, UK) were included in each run.

### 2.4. Digital Slide Scanning and Automated Image Analysis

Slides were scanned with an Aperio AT2 digital slide scanner (Leica Biosystem, Milton Keynes, UK) with a 20× lens, and the quality of the images was checked manually before the application of the digital algorithm. Automated digital image analysis was performed using the Visiopharm Integrator System (Visiopharm, Hoersholm, Denmark). A cytoplasmic algorithm from the ONCOTOPIX module (v4.2.2.0, Visiopharm, Hoersholm, Denmark) was fine-tuned for the interpretation of PRDX1 staining. As image analysis output, we used H-score, which was calculated using the following formula: [1 × (% of weakly positive cells) + 2 × (% of moderately strong positive cells) + 3 × (% strong positive cells)], where the H-score of 0–100 was generally categorized as low expression, 101–200 as intermediate expression, and 201–300 as high expression of PRDX1.

### 2.5. Cell Line Culture

Triple-negative MDA-MB-231 human breast carcinoma cell line was purchased from the European Collection of Cell Cultures (Wiltshire, UK). HCC1806 (a TNBC cell line) and MCF-10A (a non-malignant immortalized mammary cell line) were gifts from Dr. Anna Marusiak (CeNT, University of Warsaw, Warsaw, Poland). HMEC, primary human mammary epithelial cells were purchased from Life Technologies (Carlsbad, CA, USA). Cells were cultured with RPMI-1640 (MDA-MB-231, HCC1806) media (Sigma-Aldrich, St Louis, MO, USA) supplemented with 10% fetal bovine serum (FBS) (Sigma-Aldrich), 2 mM L-glutamine (Sigma-Aldrich), and 1% antibiotics (penicillin/streptomycin) (Sigma-Aldrich) in humidified atmosphere containing 5% carbon dioxide (CO_2_). MCF-10A cells were cultured in mammary epithelial basal media (MEBM, Lonza, Basel, Switzerland), containing 0.4% bovine pituitary extract (BPE), 10 ng/mL human epidermal growth factor (hEGF), 5 µg/mL human insulin, 0.5 µg/mL hydrocortisone, 30 µg/mL gentamicin, 15 µg/mL amphotericin, and 100 ng/mL cholera toxin (Sigma-Aldrich). HMEC cells were cultured in HuMEC medium supplemented with epidermal growth factor, hydrocortisone, isoproterenol, transferrin, insulin, and 50 µg/mL bovine pituitary extract, according to manufacturer protocol (Life Technologies, Carlsbad, CA, USA). All cell lines were maintained through continuous passaging and were confirmed to be free of contamination by *Mycoplasma* spp. The culture media used in this project did not contain sodium pyruvate, as this compound mediates elimination of H_2_O_2_ [28].

### 2.6. Western Blotting

Cells were seeded onto a 6-well plate at 5 × 10^5^ cells/well in an appropriate medium. After 24 h, cells were washed twice in PBS and lysed using lysis buffer supplemented with protease inhibitor cocktail (Roche, Indianapolis, IN, USA). Protein concentration was determined using the bicinchoninic acid (BCA) method (Pierce, IL, USA). Before the gel electrophoresis, samples were reduced and denatured. Equal amounts (25 µg) of total protein were loaded onto SDS-PAGE. Then, proteins were transferred onto nitrocellulose membrane followed the incubation with 10% nonfat dry milk or 5% BSA in TBS-Tween 20 for 1 h at 25 °C. Afterward, the membrane was incubated overnight at 4 °C with anti-PRDX1 antibody (cat. No. HPA007730, Sigma-Aldrich, St. Louis, MO, USA, dilution 1:1000) and anti-β-actin-HRP (cat. No. A3854, Sigma-Aldrich, dilution 1:40,000). Blots were exposed to the enhanced chemiluminescent substrate (West Femto Maximum Sensitivity Substrate Pierce/Thermo Scientific, Waltham, MA, USA) and detected using the ChemiDoc Touch imaging system (Bio-Rad Laboratories, Hercules, CA, USA).

### 2.7. Chemical Reagents

Menadione (2-methyl-1,4-naphthoquinone sodium bisulfite, cat. No. M5750, Men) and sodium L-ascorbate (cat. No. A7631, Asc) were obtained from Sigma-Aldrich (St Louis, MO, USA); the reagents were dissolved in sterile distilled water. Auranofin (AUR) was purchased from Santa Cruz Biotechnology and dissolved in DMSO.

### 2.8. Stable shRNA-Mediated Knockdown of PRDX1

Knockdown of *PRDX1* expression in breast cancer cell lines (MDA-MB-231, HCC1806) and non-malignant MCF-10A cell line by use of lentiviral-mediated shRNA method was performed as described previously [13,22].

### 2.9. In Vitro Combinations with Prooxidant Agents

The cytotoxicity of the prooxidant compounds was assessed as described previously [29]. Briefly, cells were treated with increasing concentrations of Men, Asc, or Men/Asc combination, AUR alone, or combined with either Men or Asc for 24 h. Next, the crystal violet assay was applied to determine the viability of cultured cells [29]. Drug combination studies and their synergy quantification were calculated using the Chou–Talalay method by CompuSyn software v1.0 (Combosyn, Inc., Paramus, NJ, USA) [30] as described previously [9]. The observed values in all treatment groups were normalized to untreated control. According to the obtained effects of individual drug treatment and drugs in combination, the resulting combination index (CI) for additive effect (CI = 0.9–1.1), synergism (CI < 0.9), and antagonism (CI > 1.1) was calculated [30]. Experiments were performed at least in triplicates.

### 2.10. ROS Detection

MDA-MB-231 cells were seeded 3 × 10^5^ per well in 12-well plate and allowed to grow for 24 h in FluoroBrite DMEM (Life Technologies, Carlsbad, CA, USA) medium supplemented with 10% FBS, 1% Pen/Strep, and 2 mM L-glutamine, to reduce background fluorescence. Next, the CellROX^®^ Deep Red dye (Life Technologies) was added at a final concentration of 0.5 µM, for 30 min. The digital images were acquired using the green (GFP-positive cell) and red (CellROX) fluorescence channel using Celldiscoverer 7 platform (Carl Zeiss, Oberkochen, Germany).

### 2.11. Crystal Violet Assay

First, 0.5% crystal violet (Sigma-Aldrich) in 20% methanol was added to each well and incubated for 15 min at 25 °C. Then, the plate was washed in a gentle stream of tap water and air-dried. Residual dye was diluted with 2% SDS for 30 min and mixed by orbital shaking at 300–500 rpm. The optical density of each well at 560 nm was measured with a plate reader (Asys UVM 340, Biochrom, UK).

### 2.12. Colony Formation Assay

To evaluate the long-term proliferation rate of PRDX1-downregulated MDA-MB-231 cell line, modified and control cells were plated in pre-tested appropriate densities yielding 1000 into 6-well culture plates and cultured for seven days to allow colony formation. Next, the colonies were stained with 0.5% crystal violet (Sigma-Aldrich) in 20% methanol. Digital images of the colonies were obtained using a BioRad GS-800 Calibrated Densitometer (BioRad Laboratories, Hercules, CA, USA) and further analyzed with Fiji software [31]. Colonies smaller in size than 13 square pixels were omitted from the analysis according to the digital analysis guidance for breast cancer cell lines [32]. Survival fraction was plotted as the percent of the plating efficiency of shNTC and shPRDX1 MDA-MB-231 in comparison to parental MDA-MB-231 cells. The experiment was performed in triplicates and repeated three times. Additionally, the number of cell colonies, their area, and the distribution of their size was calculated and plotted with the help of ImageJ macro PHICS (Institute of Experimental Physics, Faculty of Physics, University of Warsaw, Warsaw, Poland) [33].

### 2.13. Statistical Analysis

All statistical analysis was performed using GraphPad Prism 8 (GraphPad Software, San Diego, CA, USA). The statistical values are reported as mean ± standard error of the means (S.E.M.). The differences between groups were analyzed using Student’s *t*-test (only two groups) or one-way ANOVA test (more than two groups compared) followed by a Tukey’s honestly significant difference (HSD) post hoc test (when *p* < 0.05). A probability value of *p* < 0.05 was considered statistically significant (* *p* < 0.05; ** *p* < 0.01; *** *p* < 0.001; **** *p* < 0.0001).

## 3. Results

### 3.1. Expression of PRDX1 in TNBC

**Figure 1:**
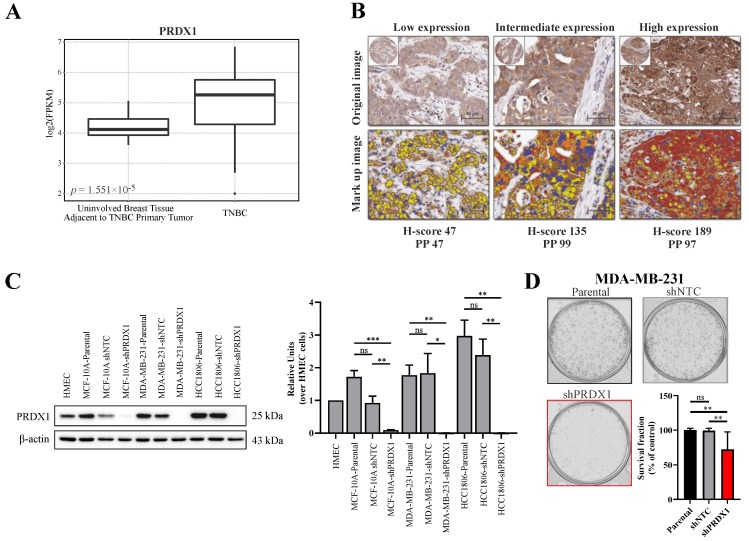
Characterization of peroxiredoxins 1 (PRDX1) expression level and its knockdown in triple-negative breast cancer. (**A**) Analysis of the expression of PRDX1 mRNA in normal (*n* = 21) and Triple-negative breast cancer (TNBC) (*n* = 42) tissues available in the analyzed NCBI Gene Expression Omnibus (GEO) database. (**B**) Representative cores from breast cancer TNBC tissue microarray (TMA), stained using anti-PRDX1 antibody via IHC approach, displaying low, intermediate, and high PRDX1 protein expression (top) and the corresponding mark-up images following application of image analysis approach (bottom). Image analysis derived corresponding H-score and percentage of positive cells (PP) is given below the images: red refers to high expression, brown is intermediate, and yellow relates to low expression. (**C**) Representative Western blotting results (left) showing knockdown of PRDX1 protein in MCF-10A, MDA-MB-231, and HCC1806 cell lines compared to parental and shNTC controls. β-actin was used as a loading control. Bands were quantified by densitometry; it was calculated as the quotient of the densitometry signal for PRDX1 band and that for β-actin and then normalized to that of the HMEC. Averaged value from three independent experiments is shown (right) (* *p* < 0.05, ** *p* < 0.01, *** *p* < 0.001, ns: not significant). (**D**) Representative images for the colony formation in MDA-MB-231 cells and the cell survival fraction (SF) calculated by clonogenic assay. The percent SF over parental control is presented as mean ± S.E.M. Data shown are cumulative results from three independent experiments repeated in triplicates. Statistical analysis was performed with one-way ANOVA followed by Tukey’s honestly significant difference (HSD) post hoc test when significance was detected (** *p* < 0.01, ns: not significant).

As *PRDX1* was reported to be upregulated in breast cancer in general [13,21], we hereby analyzed the publicly available dataset for the gene expression of *PRDX1* mRNA within the TNBC subtype as compared to non-malignant tissues. As shown in Figure 1A, based on 42 TNBC cases versus 21 uninvolved breast tissue samples that were adjacent to the TNBC primary tumors, we observed that transcripts for *PRDX1* were markedly elevated in malignant tissues when compared to the non-malignant specimens. Furthermore, we analyzed the immunohistochemical staining of TMA derived from 109 TNBC samples for the expression of PRDX1 protein. As shown in Figure 1B, PRDX1 was detected in the cytoplasm of the cancer cells with variable intensity. The distribution of the H-scores (HS) for PRDX1 expression across the whole TMA is presented in Figure S1A. To dichotomize the data into low and high expression (measured by HS), we used the logrank test for Cox’s proportional hazard models to optimize the cutoff point. The cutoff for both endpoints Distant Metastasis Free Survival (DMFS) and Breast Cancer-Specific Survival (BCSS) was the same with 64% patients assigned to the low expression group (HS < 84). A significant correlation between the high expression of PRDX1 and the two endpoints was detected: DMFS (Figure S1B) and BCSS (Figure S1C). Patients in the higher expression group experienced a higher Kaplan–Meier (KM) estimate for both DMFS (HR 0.26 [CI (0.09–0.74)], *p* = 0.006) and BCSS (HR 0.26 [CI (0.09–0.75)], *p* = 0.007).

### 3.2. Effects of Downregulation of PRDX1 in TNBC Cell Lines

To downregulate PRDX1 in TNBC cell lines (MDA-MB-231 and HCC1806) and in the non-malignant MCF-10A cell line, the shRNA-mediated knockdown approach was used, as described previously [13]. The potency of the PRDX1 protein knockdown was checked by Western blotting (Figure 1C). In the functional studies on the consequences of the PRDX1 knockdown, the results of the colony formation assay showed that the colony survival fraction was significantly reduced in the MDA-MB-231-shPRDX1 cells as compared to the controls (Figure 1D). The frequency of the colony size distribution presented in Figure S2A revealed that the MDA-MB-231-shPRDX1 cells formed smaller colonies in comparison to MDA-MB-231-shNTC or MDA-MB-231 parental cells, and that the percentage of the well area occupied by shPRDX1 cells was significantly decreased (Figure S2B). This indicates growth retardation related to the disruption of the clonogenic potential of the MDA-MB-231-shPRDX1 cells. In addition, these cells presented increased levels of oxidative stress in the steady-state when compared to shNTC controls, as shown in Figure S3 and by others [20]. This substantiates the functional dependence of TNBC cells on PRDX1, as a major antioxidant enzyme and a protector of the redox homeostasis.

### 3.3. Effects of PRDX1 Knockdown on the Susceptibility TNBC Cells to Menadione

**Figure 2:**
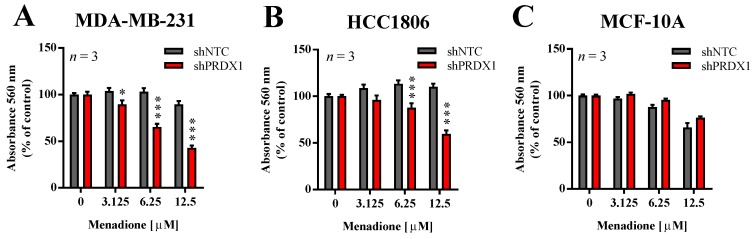
Effect of PRDX1 knockdown on Men cytotoxicity to MDA-MB-231, HCC1806, and MCF-10A cell lines. Cytotoxic effect of Men on malignant MDA-MB-231 (**A**), HCC1806 (**B**), and non-cancerous MCF-10A (**C**) cell lines is shown. Cells were treated with increasing concentrations of menadione (3–12.5 µM) for 24 h. Control cells were cultured without any reagent. At the end of treatment, the crystal violet staining was performed and reported as percent growth relative to control. An experiment was performed in triplicates and repeated three times. Statistical analysis was performed with one-way ANOVA followed by Tukey’s honestly significant difference (HSD) post hoc test when significance was detected (* *p* < 0.05, *** *p* < 0.001).

In our previous studies, we showed that the downregulation of PRDX1 in breast cancer cell lines, including TNBC cell lines, results in markedly increased toxicity of Asc [13]. In addition, He et al. reported that PRDX1 knockdown potentiates Men-induced, but not classical chemotherapy-induced, toxicity in human cervical adenocarcinoma HeLa and human lung cancer A549 cell lines, but to a lesser extent in HUVEC cells or normal fibroblasts [14]. Therefore, in this study, we evaluated the effects of PRDX1 knockdown on sensitivity to Men in two TNBC cell lines (MDA-MB-231 and HCC1806) and compared them to the non-malignant MCF-10A cell line, also of triple-negative phenotype. As shown in Figure 2, the toxicity of Men was significantly potentiated by PRDX1 knockdown only in TNBC cell lines, but not in MCF-10A cells. Indeed, the calculated EC_50_ value for Men for HCC1806-shNTC cells was 65.2 ± 8.7 µM, while for MDA-MB-231-shNTC cells it was 35.4 ± 6.1 µM, and those results were significantly different (Figure S4). However, this difference in response to Men was no longer seen in MDA-MB-231-shPRDX1 vs. HCC1806-shPRDX1 cells (Figure 2A,B), i.e., the respective EC_50_ values for HCC1806-shPRDX1 (23.0 ± 7.0 µM) and MDA-MB-231-shPRDX1 (12.0 ± 3.0 µM) (Figure S4). We previously observed a similar phenomenon in the case of TNBC cell lines response to Asc, as reported in our recent work (Figure S5 in [13]), which suggests that the mechanism of resistance to Men or Asc in TNBC cells can be indeed PRDX1-dependent. This finding corroborates the particular role of PRDX1 as a gatekeeper of redox homeostasis in TNBC cells.

### 3.4. Effects of Asc/Men Combination in TNBC Cells with Downregulated PRDX1

**Figure 3:**
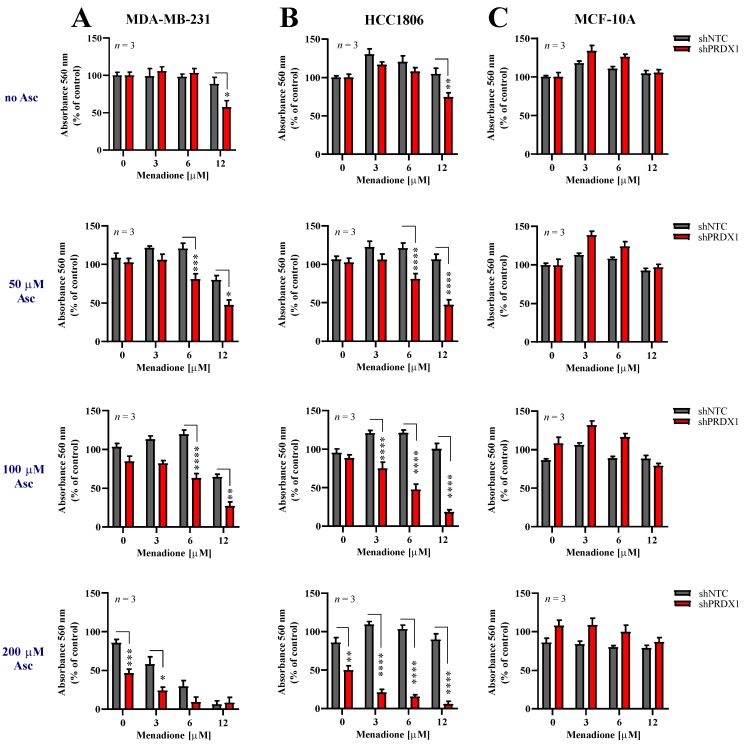
Downregulation of PRDX1 sensitizes triple-negative breast cancer cells to prooxidant agents. Cytotoxic effect of Men and Asc on malignant MDA-MB-231 (**A**) and HCC1806 (**B**) and non-malignant MCF-10A (**C**) cells with reduced expression of PRDX1 (shPRDX1) or control cells (shNTC). Cells were treated with increasing concentrations of menadione (3–12 µM) and/or sodium L-ascorbate (50–200 µM) for 24 h. For all cytotoxicity assays, control cells were cultured without any reagent. At the end of treatment, the crystal violet staining was performed and reported as percent growth relative to control. Experiments were performed in triplicates and repeated three times. Statistical analysis was performed with one-way ANOVA followed by Tukey’s honestly significant difference (HSD) post hoc test when significance was detected (* *p* < 0.05, ** *p* < 0.01, *** *p* < 0.001, **** *p* < 0.0001).

Given the information that downregulation of PRDX1 potently sensitizes TNBC cells to either Asc [29] or Men (Figure 2), we evaluated the response of two TNBC cell lines, i.e., MDA-MB-231-shPRDX1 (Figure 3A) or HCC1806-shPRDX1 (Figure 3B), or the non-malignant MCF-10A-shPRDX1 (Figure 3C) cells to the combined Asc/Men treatment as compared to the respective shNTC controls (Figure 3A–C) or parental cells (Figure S5). We noticed that both MDA-MB-231-shPRDX1 and HCC1806-shPRDX1 (Figure 3A,B, respectively) were distinctly more sensitive to incubation with Asc/Men combination as compared to the respective controls (Figure 3A and Figure S5A for MDA-MB-231 cells and Figure 3B and Figure S5B for HCC1806 cells). When the combination indexes were calculated, values below 0.9 (i.e., indicating a synergistic interaction) were seen for markedly lower concentrations of Asc and Men in the shPRDX1 cells than in the respective controls (parental and shNTC) for both MDA-MB-231 and HCC1806 cell lines (Figure S5D,E, respectively). Conversely, no amplification of Asc/Men effects by PRDX1 knockdown was detected in the non-malignant MCF-10A cell line (Figure 3C) when compared to the controls (Figure 3C and Figure S5C).

### 3.5. Effects of Auranofin on the Susceptibility of TNBC Cells to Asc/Men

**Figure 4:**
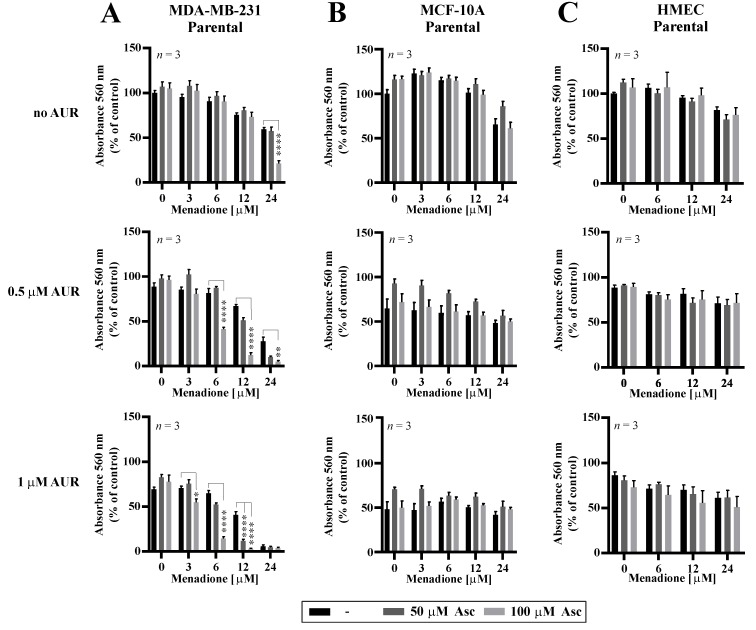
Cytotoxic effects of combinations of auranofin, menadione, and ascorbate in malignant and non-malignant cells. MDA-MB-231 (**A**) and non-cancerous MCF-10A (**B**) and HMEC (**C**) cells were treated with increasing doses of Men (3–24 µM) and L-Asc (50, 100 µM) in the absence or presence of AUR (0.5–1 µM) for 24 h. At the end of treatment, cell proliferation was determined by crystal violet staining and reported as percent growth relative to control. Mean ± S.E.M. of the three independent experiments is shown. Statistical analysis was performed with one-way ANOVA followed by Tukey’s honestly significant difference (HSD) post hoc test when significance was detected (* *p* < 0.05, ** *p* < 0.01, **** *p* < 0.0001).

Following the results of potentiated effectiveness of Asc/Men combination in TNBC cells with genetically downregulated PRDX1, we evaluated how the chemical inhibition of the PRDX1-related enzymatic system influences the response to Asc/Men in TNBC cells. Previously in our studies, we utilized several chemical inhibitors of the PRDX1-related system, such as SK053 [14], adenanthin [34], or auranofin (AUR) [13]. Out of these compounds, only AUR is a clinically applicable agent. It is also worth noting that our team and others previously reported the synergistic cytotoxic effects of a combination of AUR and Asc in B-cell malignancies [13] and TNBC [35], respectively. Therefore, in this study, we chose AUR for the combination with the Asc/Men treatment. We observed a significant amplification of the antitumor effects in the MDA-MB-231 (Figure 4A, confirmed by combination index calculations, as presented in Figure S6A) and, to the lesser extent, HCC1806 (Figure S6B) TNBC cell lines when AUR and Men/Asc were applied together, but not in the non-malignant MCF-10A cells (Figure 4B) or the telomerase-immortalized human mammary epithelial cells (HMEC, Figure 4C). Again, the calculated values of combination indexes confirm synergistic interactions in triple AUR/Asc/Men combinations (Figure S6A,C for MDA-MB-231 and HCC1806 cells, respectively).

## 4. Discussion

Despite high expectations and numerous enthusiastic reports from the preclinical studies [36], redox-modulating agents used against cancer cells have not proceeded into a standard therapeutic approach in clinical settings up to date (reviewed in [37]). A classic example of such a disappointing treatment is the combination of ascorbate and menadione (Asc/Men), highly effective in preclinical settings [4,5,6,7], but with little benefits for the patients observed in a clinical trial [6]. This situation can be caused by the fact that achieving long-lasting high concentrations of either Asc or Men is a challenging task in the human body, especially following oral administration [9,10]. Additionally, many cancers are intrinsically adapted to the hypermetabolism with elevated ROS production and to the subsequent exaggeration of oxidative stress. Thus, when treated with insufficiently high concentrations of prooxidants, these malignant cells can further adapt by increasing their cytoprotective antioxidant capacity and eventually become more resistant to the redox-targeted approaches. To combat this phenomenon, an idea of combining ROS inducers with antioxidant silencers has been proposed previously [38], and, subsequently, accumulating evidence has been gathered that such augmented prooxidant therapy (APoT, reviewed in [39]) may eventually help prooxidant treatment to enter into clinical settings as a successful, non-experimental approach. Following this notion, it became essential to identify the most pronounced adaptation mechanisms that cancers can use against a given prooxidant treatment, in order to successfully combine the inhibitors of such systems along with the prooxidants.

In the current study, based on the previous observations from our laboratory and other research teams, we set forth to study PRDX1 as a putative antioxidant enzyme responsible for resistance to the combined Asc/Men treatment. As a model, we chose TNBC—a malignancy with highly unfavorable prognosis and still awaiting effective treatment options.

In our work, we provide evidence that *PRDX1*, at both mRNA and protein levels, is markedly overexpressed in primary TNBC tumors, which remains in accordance with recent observations by Mei et al. [40]. We also present the analysis of TNBC TMA histopathological staining for PRDX1 protein concerning the survival parameters of the patients, suggesting that higher expression of PRDX1 protein can be regarded as a favorable prognosis biomarker in TNBC. This corresponds to the previous observation by O’Leary et al. in the general population of breast cancer patients [22]. In addition, correspondingly to our previous results in other subtypes of breast cancer, knockdown of PRDX1 results in inhibition of growth, as assessed in a colony formation assay, and an increase of oxidative stress in MDA-MB-231 TNBC model cell line. This provides evidence for the importance of PRDX1 protein as a protector of intracellular redox homeostasis in TNBC cells and the attractiveness of PRDX1 as a potential therapeutic target in this disease.

An open question remains whether PRDX1 protein expression can become a predictive biomarker for the response of TNBC to the prospective treatment strategies involving Asc, Men, or other prooxidant agents. The results presented hereby, along with our observations reported recently [13] suggest that the TNBC cases with natively higher expression of PRDX1 may be more resistant to prooxidant treatment. At the same time, such cases could be considered for an APoT-type treatment with prooxidants combined along with the targeting of the PRDX1-related antioxidant system. This notion, however, warrants extended investigations.

We would like to point out the “paradoxical dualism” of PRDX1 expression and its role in breast cancer, including TNBC. Our research team has been addressing this phenomenon, as discussed in our previous works [22,29]. We hypothesize that the increased presence of PRDX1, induced by the altered redox and bioenergetic homeostasis that characterize TNBC cells [41], stabilizes to some extent the redox homeostasis in breast cancer cells, decreases further spontaneous mutagenesis, and slows down its progression into more aggressive forms, and thus it correlates with a more favorable prognosis. However, at the same time, PRDX1 does protect the malignant cells from immediate death due to oxidative stress, and thus promotes the cancerous growth, which suggests that the inhibition of PRDX1 in breast cancer could be a potential therapeutic approach in this disease, especially when combined with prooxidant therapies, such as Asc/Men.

Generally, the idea of targeting PRDX1 in breast cancer has been proposed previously, and that notion originates from the observation that PRDX1 can inhibit H_2_O_2_-induced cell death in mammary carcinoma cells [42]. Our current results are strongly supportive of the validity of this approach by showing that genetic silencing of PRDX1 in TNBC cell lines amplifies the efficacy of one of the most promising prooxidant approaches, i.e., the combination of Asc and Men (Figure 3 and Figure S5D,E). The difficulty of PRDX1 targeting in cancer in the clinical settings is related to the fact that chemical agents known to inhibit PRDX1, such as adenanthin [34,43], AI-44 [44], or frenolicin B [45], or the PRDX1-related antioxidant enzymatic system, such as SK053 [14] or AUR [13], are in most cases fairly unselective and/or do not possess satisfactory pharmacokinetics. The exception for the latter is AUR, the clinically approved drug primarily used in rheumatology and suggested in numerous other indications [46]. AUR is known to inhibit, among others, selenoprotein thioredoxin reductase (TXNR) [47], the inhibition of which blocks PRDX1 reduction and hydrogen peroxide removal. Accordingly, AUR has been shown to hamper the functionality of TXN-dependent PRDX1 and PRDX3 enzymes in MDA-MB-231 cells [35]. For that reason, AUR is also considered for repurposing as an anticancer drug [48]. The results presented in the current study support this notion—we observed AUR to potently amplify the cytotoxic effects of Asc/Men combination in TNBC, but not in non-malignant mammary cell lines. This tumor-selective effect encourages the further evaluation of the triple AUR/Asc/Men combination as an anticancer approach in TNBC, including the potential evaluation in humans, as all these agents have already been tested in clinical settings. It is important, however, to take into consideration the fact that chemical inhibition by AUR is expected to provide a broader and more complex spectrum of changes into the cellular metabolic machinery than a specific genetic disruption of PRDX1 expression, as discussed above. This can explain some differences in the response of the cell lines studied in the current work to the genetic targeting of PRDX1 versus AUR.

Another outstanding question is how targeting PRDX1 or PRDX1-related antioxidant system would modify the response of cancer cells to transiently high (i.e., at mM ranges for Asc) concentrations achieved by intravenous application of either Asc or Men, and especially both compounds applied together. Indeed, in our previous work [29], we tested the continuous incubation of MCF-7 cells with the concentrations of Asc up to 1.6 mM when used alone, and we found a potent toxicity (approximately 50%) of Asc alone used in such concentrations. For the current work, we considered such effects too potent for the combination studies, and therefore we utilized lower concentrations of Asc. However, it remains of interest how the TNBC cells with targeted PRDX1 would respond to a “pulse” of high concentrations of Asc, Men or Asc/Men combination, mimicking the effects of intravenous application of Asc. This issue warrants further investigations.

## 5. Conclusions

In summary, our work provides further evidence for the validity of the augmented prooxidant therapy anticancer approach, where ROS-inducing agents are combined with the inhibitors of respective, preferably cancer-specific, cellular defenses. Our study also provides support for the exceptional role of PRDX1 protein as well as the PRDX1-related antioxidant system as central for maintaining redox homeostasis in TNBC cells. This encourages further research towards finding the specific inhibitor for PRDX1 and evaluating PRDX1 protein expression in TNBC as a potential predictive marker for the response to prooxidant therapies.

## Supplementary Materials

The following are available online at https://www.mdpi.com/2076-3921/9/4/320/s1, **Figure S1**: The RATHER TNBC TMA cohort analysis. The PRDX1 expression data (using the measure of H-score in) was assessed with a cut-off optimized by maximum separation using the logrank test. The cutoff for both end-points (DMFS and BCSS) was the same (**A**), with 64% of patients assigned to the lower expression group (HS < 84). Patients in the higher expression group experienced a higher KM estimate both DMSF (**B**) (HR 0.26 [CI (0.09–0.74)], *p* = 0.006) and BCSS (**C**) (HR 0.26 [CI (0.09–0.75)], *p* = 0.007). **Figure S2**: Clonal growth characterization of parental, shNTC, and shPRDX1 MDA-MB-231 cells. (**A**) The statistical distribution of colonies in relation to their size formed seven days after seeding 1 × 10^3^ of parental, shNTC, or shPRDX1 MDA-MB-231 cells. Histograms depict mean frequency of the colonies similar in size, and the red line represents Weibull fitting. ImageJ analysis was performed with Plot Histograms of Colony Size (PHICS) macro. (**B**) The mean percent of the well area occupied by cell colonies formed by parental, shNTC, or shPRDX1 MDA-MB-231 cells. Values represent the cumulative data from three independent experiments ± S.E.M. Statistical analysis was performed with one-way ANOVA followed by Tukey’s honestly significant difference (HSD) post hoc test when significance was detected (*** *p* < 0.001). **Figure S3**: Cellular ROS levels assessed in live PRDX1-knockdown and shNTC MDA-MB-231 cells by CellROX Deep Red reagent. Cells were cultured for 24 h, then incubated in the presence of CellROX Deep Red for 30 min. Representative bright-field images, fluorescence images (GFP-positive cell and red-CellROX), and merge are shown. Scale bar: 100 μm. Fluorescence intensity was assessed using Celldiscoverer 7 platform (Zeiss). **Figure S4**: EC_50_ of Men for PRDX1 knockdown on MDA-MB-231 and HCC1806 cell lines compared to shNTC controls (left). EC_50_ was calculated for a range of Men concentration (0–200 µM) (right). The differences between groups were analyzed using Student’s *t*-test (only two groups) (* *p* < 0.05, ** *p* < 0.01, *** *p* < 0.001, ns: not significant). **Figure S5**: The prooxidant cytotoxicity of the combination of Men and Asc on triple-negative breast cancer and normal cells. The effect of Men and Asc on malignant parental MDA-MB-231 (**A**) and HCC1806 (**B**) cells and non-malignant parental MCF-10A cells (**C**). Cells were treated with increasing concentrations of menadione (3–12 µM) and/or sodium L-ascorbate (50–200 µM) for 24 h. For all cytotoxicity assays, control cells were cultured without any reagent. At the end of treatment, the crystal violet staining was performed and reported as percent growth relative to control. Experiments were performed in triplicates and repeated three times. Statistical analysis was performed with one-way ANOVA followed by Tukey’s honestly significant difference (HSD) post hoc test when significance was detected (** *p* < 0.01, *** *p* < 0.001). The combination index (CI) calculated by the Chou–Talalay method was used to determine drug interaction (**D**,**E**). The CI is reported at different doses of Men and Asc, as indicated in tables. CI values < 0.9 suggest synergism. **Figure S6**: Cytotoxic effects of combinations of auranofin, menadione, and ascorbate in TNBC cell lines. The combination index (CI) calculated by the Chou–Talalay method was used to determine drug interaction ((**A**,**C**) MDA-MB-231 and HCC1806, respectively). The CI is reported at different doses of prooxidants and AUR, as indicated in the graph (left side) and table (right side). The average values of CI were calculated from three independent experiments. CI values < 0.9 suggest synergism. (**B**) HCC1806 cells were treated with increasing doses of AUR (0.5–2 µM) in the absence or presence of Men (3–24 µM) and L-Asc (50, 100 µM) for 24 h. At the end of treatment, cell proliferation was determined by crystal violet staining and reported as percent growth relative to control. Mean ± S.E.M. of the three independent experiments is shown.
