# Supplementary material for: Triple Combination of Ascorbate, Menadione and the Inhibition of Peroxiredoxin-1 Produces Synergistic Cytotoxic Effects in Triple-Negative Breast Cancer Cells
Source: Antioxidants (Basel). 2020 Apr 16;9(4):320. doi: 10.3390/antiox9040320 (PMC7222372; doi:10.3390/antiox9040320)
Supplement: Supplementary file 1 [file antioxidants-09-00320-s001.pdf]

Supplementary Figures and Figure Legends

Supplementary Figure S1

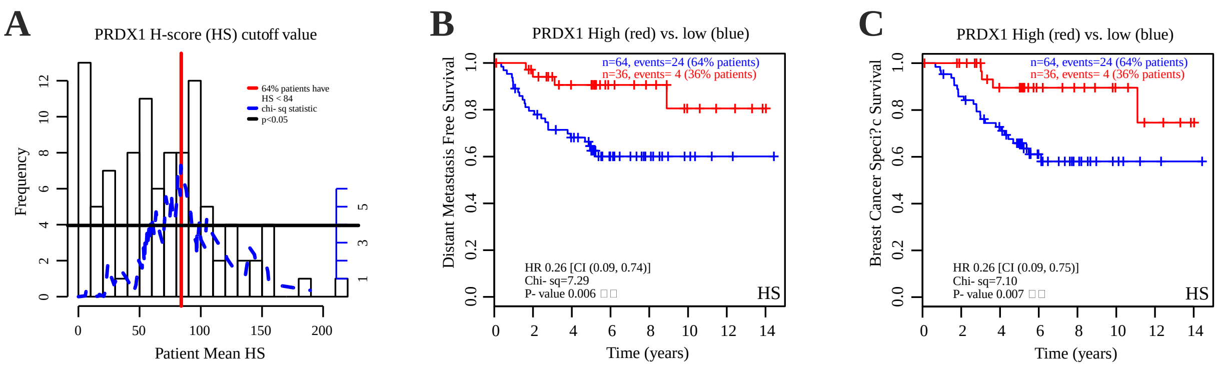

Supplementary Figure S1. The RATHER TNBC TMA cohort analysis. The PRDX1 expression data (using the measure of H-score in) was assessed with a cut-off optimized by maximum separation using the logrank test. The cutoff for both end-points (DMFS and BCSS) was the same (**A**), with 64% of patients assigned to the lower expression group (HS < 84). Patients in the higher expression group experienced a higher KM estimate both DMSF (**B**) (HR 0.26 [CI (0.09–0.74)], $p = 0.006$) and BCSS (**C**) (HR 0.26 [CI (0.09–0.75)], $p = 0.007$).

Supplementary Figure S2

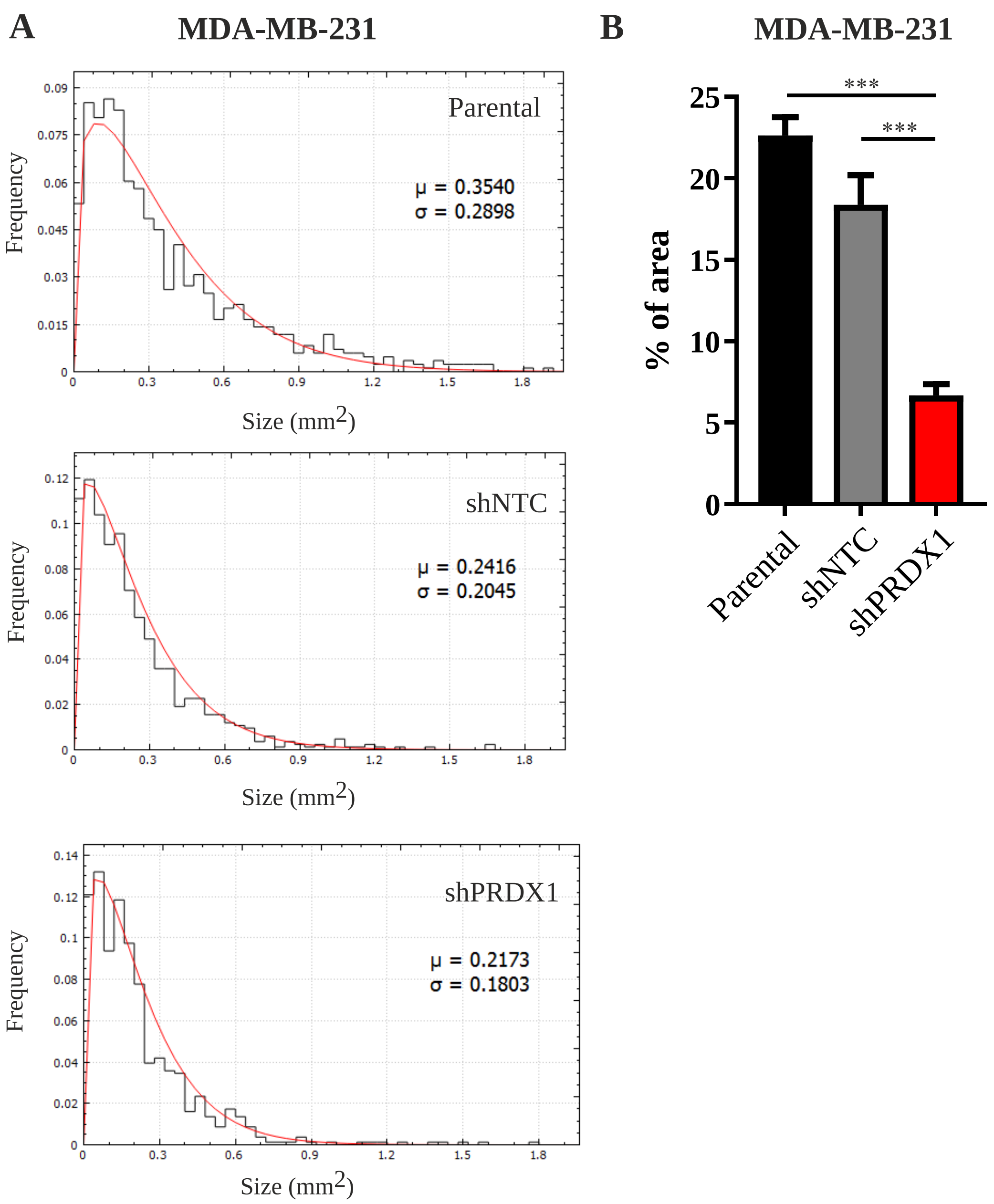

Supplementary Figure S2. Clonal growth characterization of parental, shNTC and shPRDX1 MDA-MB-231 cells. (**A**) The statistical distribution of colonies in relation to their size formed seven days after seeding 1 × 10^3 of parental, shNTC, or shPRDX1 MDA-MB-231 cells. Histograms depict mean frequency of the colonies similar in size, and the red line represents Weibull fitting. ImageJ analysis was performed with Plot Histograms of Colony Size (PHICS) macro. (**B**) The mean per cent of the well area occupied by cell colonies formed by parental, shNTC, or shPRDX1 MDA-MB-231 cells. Values represent the cumulative data from three independent experiments ± S.E.M. Statistical analysis was performed with one-way ANOVA followed by Tukey's honestly significant difference (HSD) post hoc test when significance was detected (***$p < 0.001$).

Supplementary Figure S3

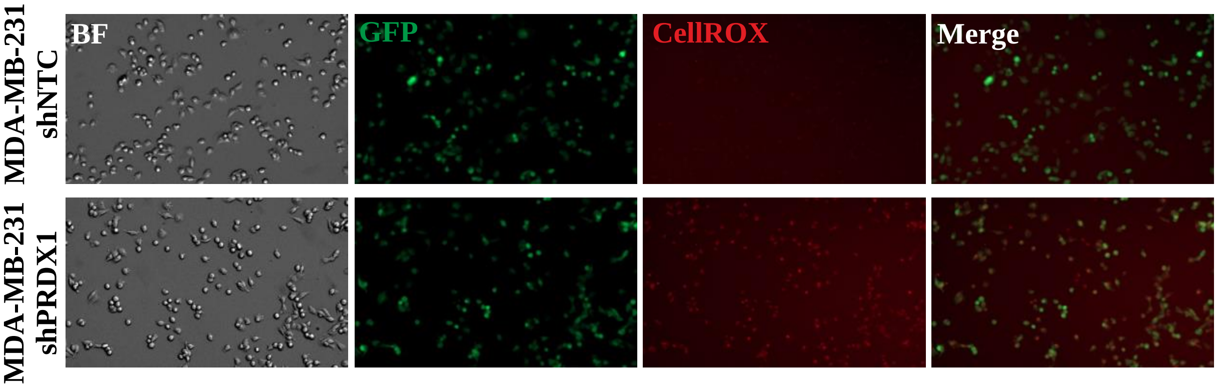

Supplementary Figure S3. A. Cellular ROS levels assessed in live PRDX1-knockdown and shNTC MDA-MB-231 cells by CellROX Deep Red reagent. Cells were cultured for 24 hours, then incubated in the presence of CellROX Deep Red for 30 mins. Representative bright-field images, fluorescence images (GFP-positive cell and red-CellROX), and merge are shown. Scale bar: 100 µm. Fluorescence intensity was assessed using Celldiscoverer 7 platform (Zeiss).

Supplementary Figure S4

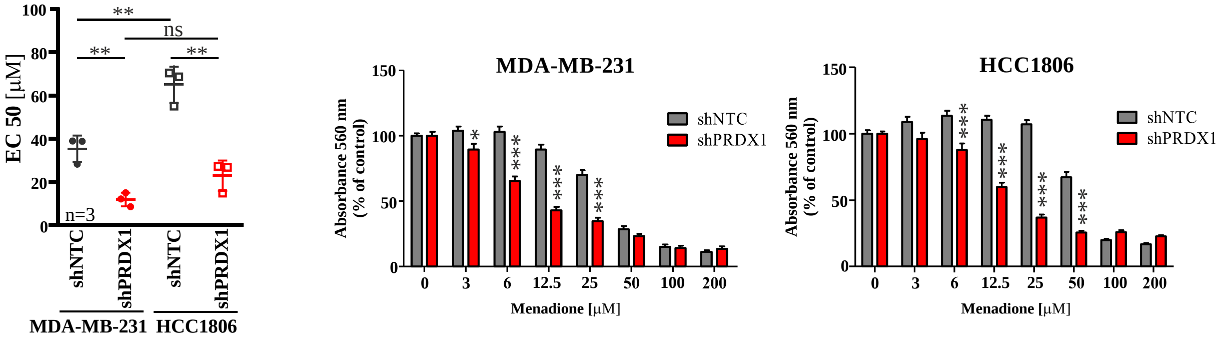

Supplementary Figure S4. EC50 of Men for PRDX1 knockdown on MDA-MB-231 and HCC1806 cell lines compared to shNTC controls (left panel). EC50 was calculated for a range of Men concentration (0–200 µM) (right panel). The differences between groups were analyzed using Student's *t*-test (only two groups) (*$p < 0.05$, **$p < 0.01$, ***$p < 0.001$, ns: not significant).

Supplementary Figure S5

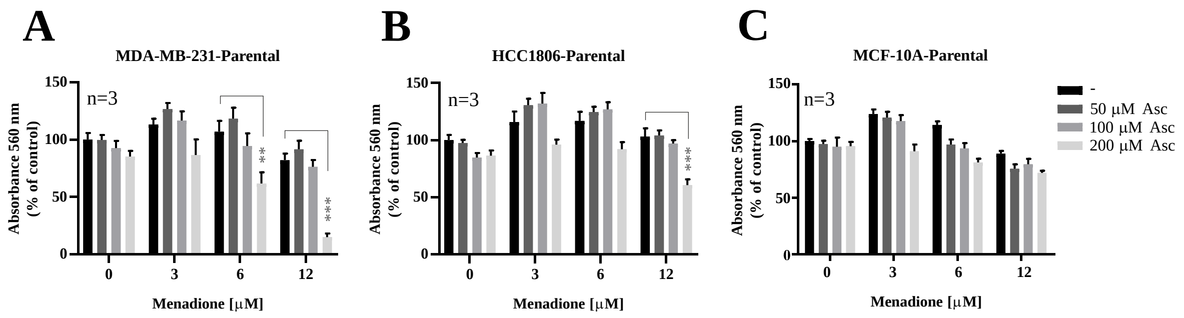

D

Combination Index (CI)	MDA-MB-231								
	Parental			shNTC			shPRDX1		
**Men [µM]**	**3**	**6**	**12**	**3**	**6**	**12**	**3**	**6**	**12**
**50 µM Asc**	4.04	6.97	1.57	4.01	2.58	0.70	3.96	0.81	0.49
**100 µM Asc**	5.17	1.85	0.80	4.80	8.02	0.46	1.08	0.84	0.51
**200 µM Asc**	1.35	0.69	0.22	0.44	0.24	0.27	0.81	0.58	0.57

E

Combination Index (CI)	HCC1806								
	Parental			shNTC			shPRDX1		
**Men [µM]**	**3**	**6**	**12**	**3**	**6**	**12**	**3**	**6**	**12**
**50 µM Asc**	12.7	15.2	20.2	3.55	6.07	11.1	7.00	0.87	0.25
**100 µM Asc**	22.9	25.4	4.12	4.59	7.11	12.1	1.04	0.53	0.25
**200 µM Asc**	7.29	2.96	0.23	6.66	9.18	1.29	0.38	0.31	0.15

Supplementary Figure S5. The prooxidant cytotoxicity of the combination of Men and Asc on triple-negative breast cancer and normal cells. The effect of Men and Asc on malignant parental MDA-MB-231 (**A**) and HCC1806 (**B**) cells and non-malignant parental MCF-10A cells (**C**). Cells were treated with increasing concentrations of menadione (3–12 µM) and/or sodium L-ascorbate (50–200 µM) for 24 h. For all cytotoxicity assays, control cells were cultured without any reagent. At the end of treatment, the crystal violet staining was performed and reported as percent growth relative to control. Experiments were performed in triplicates and repeated three times. Statistical analysis was performed with one-way ANOVA followed by Tukey's honestly significant difference (HSD) post hoc test when significance was detected (**$p < 0.01$, ***$p < 0.001$). The combination index (CI) calculated by the Chou–Talalay method was used to determine drug interaction (**D, E**). The CI is reported at different doses of Men and Asc, as indicated in tables. CI values < 0.9 suggest synergism.

Supplementary Figure S6

A

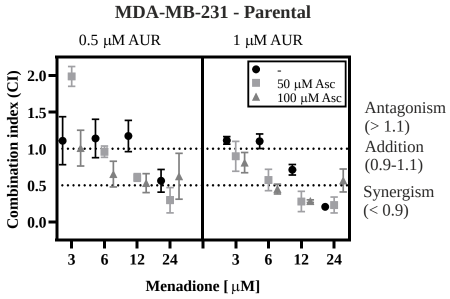

MDA-MB-231-Parental		0 Asc		50 µM Asc		100 µM Asc	
**AUR [µM]**	**Men [µM]**	**Mean**	**SEM**	**Mean**	**SEM**	**Mean**	**SEM**
**0.5**	**3**	1.11	0.33	1.99	0.14	1.01	0.24
	**6**	1.14	0.26	0.96	0.08	0.65	0.18
	**12**	1.17	0.21	0.61	0.05	0.53	0.13
	**24**	0.56	0.15	0.30	0.17	0.62	0.31
**1**	**3**	1.11	0.05	0.90	0.20	0.81	0.14
	**6**	1.10	0.10	0.57	0.15	0.45	0.07
	**12**	0.71	0.07	0.28	0.14	0.28	0.02
	**24**	0.21	0.02	0.23	0.11	0.57	0.16

B

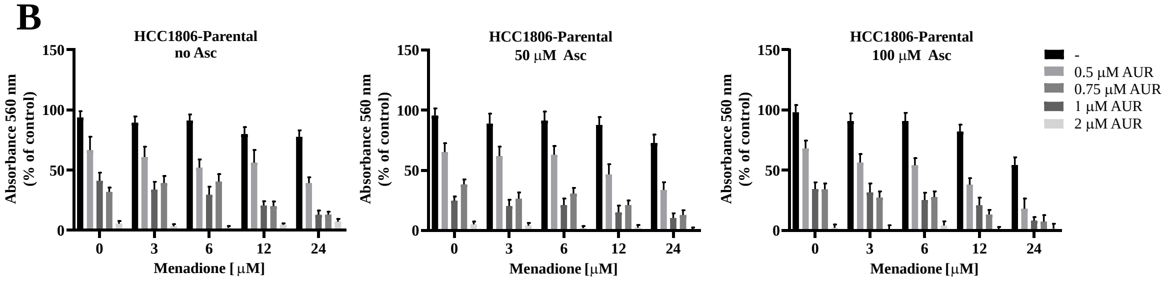

C

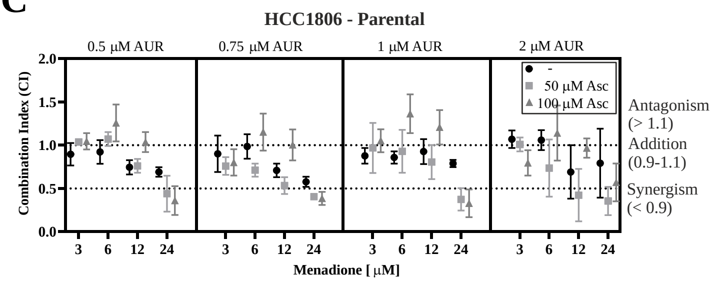

HCC1806		0 Asc		50 µM Asc		100 µM Asc	
**AUR [µM]**	**Men [µM]**	**Mean**	**SEM**	**Mean**	**SEM**	**Mean**	**SEM**
**0.5**	**3**	0.90	0.13	1.04	0.02	1.05	0.09
	**6**	0.92	0.14	1.07	0.08	1.26	0.21
	**12**	0.75	0.08	0.76	0.08	1.04	0.12
	**24**	0.69	0.06	0.44	0.21	0.36	0.17
**0.75**	**3**	0.90	0.21	0.76	0.10	0.80	0.15
	**6**	0.99	0.14	0.71	0.07	1.15	0.21
	**12**	0.71	0.08	0.53	0.10	1.00	0.18
	**24**	0.58	0.06	0.41	0.02	0.39	0.08
**1**	**3**	0.88	0.09	0.97	0.29	1.05	0.13
	**6**	0.86	0.07	0.93	0.25	1.36	0.22
	**12**	0.93	0.14	0.81	0.20	1.21	0.20
	**24**	0.79	0.04	0.37	0.13	0.33	0.16
**2**	**3**	1.07	0.10	1.01	0.08	0.80	0.15
	**6**	1.06	0.12	0.74	0.33	1.14	0.32
	**12**	0.69	0.31	0.42	0.30	0.97	0.11
	**24**	0.79	0.40	0.36	0.16	0.57	0.22

Supplementary Figure S6. Cytotoxic effects of combinations of auranofin, menadione, and ascorbate in TNBC cell lines. The combination index (CI) calculated by the Chou-Talalay method was used to determine drug interaction (**A** and **C** for MDA-MB-231 and HCC1806, respectively). The CI is reported at different doses of prooxidants and AUR, as indicated in the graph (left side) and table (right side). The average values of CI were calculated from three independent experiments. CI values < 0.9 suggest synergism. **B**. HCC1806 cells were treated with increasing doses of AUR (0.5 -2 µM) in the absence or presence of Men (3–24 µM) and L-Asc (50, 100 µM) for 24 h. At the end of treatment, cell proliferation was determined by crystal violet staining and reported as percent growth relative to control. Mean ± S.E.M. of the three independent experiments is shown.
